# Dietary deoxynivalenol does not affect mineral element accumulation in breast and thigh muscles of broiler chicken

**DOI:** 10.1007/s12550-017-0306-x

**Published:** 2018-01-08

**Authors:** Manfred Sager, Annegret Lucke, Khaled Ghareeb, Manoochehr Allymehr, Qendrim Zebeli, Josef Böhm

**Affiliations:** 1 Austrian Agency for Health and Food Safety, Special Investigations in Element Analysis, Spargelfeldstrassse 191A, 1220 Vienna, Austria; 2 Institute of Animal Nutrition and Functional Plant Compounds, Department for Farm Animals and Veterinary Public Health, University of Veterinary Medicine Vienna, Veterinärplatz 1, A-1210 Vienna, Austria; 3 Faculty of Veterinary Medicine, South Valley University, Qena, 83523 Egypt; 4 Faculty of Veterinary Medicine, Urmia University, Urmia, Iran

**Keywords:** Elements, Broilers, Deoxynivalenol, Breast, Thigh

## Abstract

Deoxynivalenol (DON), a well-known contaminant of feed, can have negative effects on gut permeability and function in poultry, which then could affect major and trace element content of the broilers’ breast and thigh muscles, and ultimately reduce meat quality. To study this hypothesis, DON-contaminated diet was fed to broiler chicks. Two groups of birds were housed in metabolic cages with free access to water and feed, with or without DON (10 mg/kg). After 5 weeks, birds were dissected and samples of the breast and thigh muscles, feed and droppings were analysed for five macro (Ca, K, Mg, Na, and P) and ten micro elements (Al, Cr, Cu, Fe, Mn, Li, Mo, Ni, Pb, Rb, and Zn) by inductively coupled plasma optical emission spectrometry (ICP-OES) or inductively coupled plasma mass spectrometer (ICP-MS) methods. In both groups, increased (*p* < 0.05) concentrations of Ca Na, Fe, Mn, and Zn were found in thigh muscles compared with the breast, whereas the concentrations of Mg, P, and Rb were higher in the breast muscles. DON had no effect on the elemental contents of the broilers’ breast and thigh muscles. In conclusion, DON at a level of 10 mg/kg feed to broiler chicken over of 5 weeks did not alter the macro or micro element composition in muscle meat.

## Introduction

It is known that chickens have a higher tolerance to DON than other animals and are frequently exposed to DON through their cereal-rich diet (Ghareeb et al. 2015; Osselaere et al. 2013; Eriksen and Pettersson 2004). However, low and moderate levels of DON contamination are responsible for various immunological modulations and effects on the gut health and inflammation of chickens (Ghareeb et al. 2013, 2015; Osselaere et al. 2013). Gut inflammation due to DON feeding leads to impairment of nutrient absorption as a result of alteration in the intestinal histology (Awad et al. 2006a, b) and reduction of the intestinal barrier function (Awad et al. 2004, 2005, 2011). An increased nutrient digestibility has been reported due to feeding of DON contaminated feed (Dänicke et al. 2003, 2007a) without affecting the level of minerals (magnesium and inorganic phosphate). Furthermore, the results of Dänicke et al. (2003, 2007a) suggested the positive impact of DON contamination in the feed of broiler chickens due to lowering the intestinal viscosity which was also found in turkeys (Dänicke et al. 2007b) and ducks (Dänicke et al. 2004) resulting in a higher nutrient digestibility. The authors also proposed the negative adverse effects of Fusarium mycotoxin contamination of poultry feed on poultry health and performance. In a recent investigation, DON was shown to reduce the mRNA expression of Zinc (ZnT)-1 transporter gene in the intestines of chickens (Antonissen et al. 2015). Moreover, a study performed by our team showed that either higher or lower level of DON decreased the concentration of calcium and potassium in the blood of chickens (Yunus and Böhm 2013). However, the quality of chicken meat with DON-contaminated diet is poorly investigated. Therefore, an important question arises whether DON feeding might alter the mineral element contents of chicken meat. In one hand, this is important from the nutritional point of view especially regarding the concentration of essential minerals in broiler meat. On the other hand, potential accumulation of heavy metals caused by changes in gut permeability after DON exposure is of interest from food safety perspective. As a consequence, the present experiment was conducted to investigate the impact of feeding high-dosed DON (10 mg DON/kg feed) on the concentrations of 15 mineral elements in the breast and thigh muscles of broiler chickens.

## Material and methods

The animal experiments approved by the institutional ethics committee under the licence number 68.205/0062-WF/V/3b/2015. All husbandry practices and euthanasia were performed according to national and European regulations and with full consideration of animal welfare. Indeed, the birds were fasted for 1 h and then anaesthetized by injection of a single dose of thiopental (50–100 mg/kg BW) into the wing vein and subsequently killed by cutting of the jugular vein.

### Birds’ diet and housing

One-day-old male broiler chicks (Ross 308) were obtained from a commercial hatchery and randomly allocated to two groups: (a) control group (*n* = 4) was fed with a basal commercial broiler diet (Biomin GmbH, Austria) and (b) DON group (*n* = 5) was fed with a basal diet contaminated with pure DON (Biomin GmbH, Austria) in a dose of 10 mg DON/kg feed. The present study is a part of a larger experiment (Lucke et al. 2017) with the same ingredients and nutrient composition of basal diet. The concentrations of nutrients were formulated according to the guidelines of the German society of animal nutrition (GfE 1999) for broiler feeding. The contaminated DON diet was prepared by mixing purified DON culture material (Biomin GmbH, Austria) to inulin in a rate of 0.03 to 0.20% to reach the required DON concentration in the premix. Inulin was used as a carrier to ensure the homogenous distribution of DON. The following premix was subsequently mixed by including the total of 250 g basal premix and mixed with 750 g of the control feed to produce the premix of contaminated diet. The latter premix was then mixed with the final feed amount to produce an average concentration of 0.16 mg DON/kg feed for control diet and 9.45 mg DON/kg feed for DON-contaminated diet (10 mg DON/kg feed), respectively. Representative samples from control and DON-contaminated diet were analysed for DON concentration with HPLC-MS/MS by Romer Labs Diagnostic GmbH (34030 Tulln, Austria). Birds were housed in metabolic cages with free access to water and feed for 5 weeks. Housing conditions including temperature and photoperiod followed standard commercial recommendation: temperature was 35 °C during the first week and reduced to 23 °C for the rest days of the experiment. Additionally, 23 h lighting was provided for birds during the first week, reduced to 22 h for the following 2 days and further reduced to 20 h for the rest of the period of experiment.

### Collection of samples

Representative samples of grower feed, chicken droppings at 5 weeks, and the breast and thigh muscles of four birds per dietary group after slaughtering were collected, wrapped in plastic bags, and stored at − 20 °C. Homogenised muscle samples were lyophilized before the element analysis performed by ICP-MS or ICP-OES.

### Element analysis of feed, dried chicken droppings, and the breast and thigh muscles

In total, 15 macro and micro elements (Ca, K, Mg, Na, P, Al, Cr, Cu, Fe, Mn, Mo, Ni, Pb, Rb, and Zn) were analysed with an analytical method as described earlier (Sager 2005). Briefly, 0.3 g of freeze-dried sample was digested with 65% of 3.9 ml HNO3 suprapur (Merck Art. 441) in closed PTEE tubes by microwave heating (MLS GmbH, D-88299 Leutkirch) and then ultrapure water was added up to 25 ml. Ca, K, Mg, Na, P, Al, Cr, Cu, Fe, Li, Mn, Mo, Pb, and Zn were measured by an ICP-OES (Perkin Elmer-Optima 3000 XL) with the following dilutions: 1 + 9, 2 +5, and non-diluted. Pb and Rb were measured using an inductively coupled plasma mass spectrometer (ICP-MS; Perkin Elmer–SCIEX Elan DRC II) with a dilution of 1 + 9 and indium was used as an internal standard. The individual muscle samples from the two dietary groups, one sample from each diet and one sample from droppings of each group, were analysed twice for each element. Two blanks were run within each series. Results were ascertained by running ALVA-ring test materials (feeds, plants). The detection limits (Table 1) were calculated from the differences between the instrument zero and the blank digests obtained within the six series of measurement. To ensure the validity of results, the operators of the analytical lab have regularly taken part in ring tests of Wageningen Agricultural University (results.wepal@wur.nl, code WELE-136), in ring tests of the National organisation ALVA, and in case of Pb in food items acted as the Austrian National Reference Laboratory (JRC-IRMM-CRL-HEAVYMETAL@ec.europa.eu), without producing outliers.

**Table 1:** The detection limits of micro and macro elements analysed in experimental samples

Element	Detection limit
Macro-element (g/kg)	
Ca	0.01
K	0.01
Mg	0.002
Na	0.005
P	0.01
Micro-element (mg/g)	
Al	0.25
Cr	0.20
Cu	0.20
Fe	3.7
Mn	0.13
Mo	0.002
Ni	0.001
Pb	0.001
Rb	0.5
Zn	1.8

### Calculations and statistics

The average value of the two measurements for each element for the individual sample of feed and droppings was calculated and subsequently the ratio of element content in droppings to feed. To investigate the impact of dietary DON on the element composition of chicken muscles in addition to evaluate the difference between breast and thigh muscles, a statistical program, SPSS (version 24) was used. Kolmogorov-Smirnov test was firstly applied to investigate the normality of the data followed by the Mann-Whitney test to evaluate the difference between the DON group and their control counterparts for the element composition of their muscles. Additionally, the difference between the two muscles was also measured by the Mann-Whitney test. Probability (*P*) value of < 0.05 was considered significant.

## Results and discussion

The ratio of the amount of element in the droppings to its amount in the feed for control and DON-fed birds (Table 2) can be used as an indicator for the net result of mineral absorption and endogenous excretion in relation to feed concentration. This ratio was numerically decreased when DON was added to the feed of broiler chicken for micro or macro elements suggesting that DON may reduce the digestibility and absorption of elements. It was shown that DON in chicken feed increased the apparent protein digestibility and the net protein utilisation without change in the level of magnesium and inorganic phosphate in the blood (Dänicke et al. 2003). The dual character of Fusarium contamination of broiler cereal grains included the positive nutritional impacts such lowering the intestinal viscosity and higher nutrient digestibility and the negative toxic effects on health and performance (Dänicke et al. 2007a, b). The authors found also that Fusarium mycotoxin infection to cereal grains of broilers resulted in physicochemical alterations of grains and increased the enzymatic activity which improved the digestibility of nutrients. In the present study, DON was added to the diet as an artificial contamination which may explain the lower ratio of elements in droppings to feed as an indication for the net absorption and excretion for birds fed with DON compared with controls (Table 2). The difference between the findings in the current experiment and the results of Dänicke et al. (2003, 2007a, b) may contribute to explain the difference between effects of natural and artificial DON contamination of poultry feed.

**Table 2:** The concentration of mineral elements in broiler control feed, DON-contaminated feed, air-dried droppings, and proportion of the excretion of mineral elements (ratio of content in dried droppings to the content in respective feed)

Element	Feed		Droppings		Ratio (droppings/feed)	
	Control	DON	Control	DON	Control	DON
Macro elements (g/kg dry matter)						
Ca	11.35	14.04	22.1	18.5	1.94	1.32
K	8.35	8.93	24.8	18.4	2.98	2.06
Mg	1.70	1.87	5.09	4.46	2.98	2.39
Na	1.66	0.88	2.15	2.01	1.29	2.28
P	6.06	8.31	13.4	11.9	2.20	1.44
Micro elements (mg/kg dry matter)						
Al	159	170	602	531	2.80	3.13
Cr	0.36	0.93	7.32	5.48	20.33	5.89
Cu	36.7	36.3	117	84	3.18	2.32
Fe	243	466	1011	915	4.16	1.96
Mn	83	109	376	365	4.51	3.36
Mo	2.49	2.66	7.71	5.95	3.09	2.24
Ni	1.47	1.90	3.77	3.90	2.56	2.05
*Pb*	0.15	0.18	0.62	0.50	4.13	2.77
*Rb*	5.04	5.54	14.07	9.33	2.79	1.68
Zn	80.2	88.1	275	217	3.43	2.46

Elements given in italics have been derived from ICP-MS measurements, all other elements were analysed by ICP-OES method

Furthermore, DON contamination did not alter (*p* > 0.05) the mineral elements of the breast and thigh muscles (Table 3) suggesting that DON in a dose of 10 mg/kg feed had no impact on the meat quality of broilers in the terms of element concentration of muscles. Within the same direction of our results, Dänicke et al. (2003) found that DON in the feed of broilers did not impact the concentration of magnesium and inorganic phosphate. This result is not online with other reports related to element absorption and their concentration in the blood. In the opposite direction, magnesium concentration in the blood of broiler was reduced after DON feeding (3.4 and 8.3 mg of DON/kg diet) (Faixová et al. 2010). Furthermore, Yunus and Böhm (2013), Katarina et al. (2011) and found that DON in broiler feed reduced the concentrations of calcium and potassium in the blood and Antonissen et al. (2015) showed a decrease in the mRNA expression of Zinc (ZnT)-1 transporter gene in the intestine under diet contaminated with DON and fumonisins. To our knowledge, the present study is the first to investigate the effect of DON in broiler feed on elemental composition of broiler muscles. The results reported in the current experiments may open a new area of research regarding the meat quality under diet contaminated with mycotoxin deoxynivalenol. Chemical composition of meat as moisture, dry matter, crude protein, fat, ash, amino acid, elements, and vitamins are the important parameters of the meat quality measurement (Sarsenbek et al. 2013). In this scenario, further experiments are required to investigate the effect of DON in the feed on bioavailability of elements and their accumulation in muscles in an experimental design allowing collection of droppings from individual birds and consequently, a statistical analysis can be performed giving an exact interpretation. On the other hand, the impact of DON on the meat quality parameters including elements as a part of chemical composition of meat needs to be addressed in the future research.

**Table 3:** The difference between breast and thigh muscles in the concentration of mineral elements whereas DON had no effect

Elements	Breast muscle (*n* = 9)			Thigh muscle (*n* = 9)			Control group (*n* = 4)			DON group (*n* = 5)		
	Median	Maximum	Minimum	Median	Maximum	Minimum	Median	Maximum	Minimum	Median	Maximum	Minimum
Macro elements (g/kg)												
Ca	0.140^b^	0.162	0.105	0.186 ^a^	0.197	0.156	0.150	0.197	0.105	0.162	0.196	0.132
K	13.800	14.952	12.168	13.361	14.668	12.099	13.503	14.650	12.168	13.800	14.952	12.099
Mg	1.140^a^	1.205	1.016	0.981^b^	1.085	0.829	1.075	1.205	0.928	1.081	1.171	0.829
Na	1.271^b^	1.472	1.087	2.004^a^	2.491	1.807	1.582	2.491	1.087	1.807	2.372	1.206
P	9.392^a^	9.696	7.935	7.856^b^	9.432	6.125	8.774	9.432	7.080	9.226	9.696	6.125
Micro elements (mg/kg)												
Al	1.32	5.52	0.23	0.83	2.10	0.60	1.43	5.52	0.62	0.71	1.97	0.23
Cr	0.25	0.29	0.20	0.13	0.30	0.21	0.23	0.29	0.20	0.24	0.30	0.01
Fe	11.13^b^	12.55	0.85	18.95^a^	23.31	7.08	8.28	23.31	0.85	12.55	21.01	9.14
Mn	0.39^b^	0.41	0.28	0.55^a^	0.75	0.40	0.44	0.75	0.28	0.40	0.73	0.36
Mo	0.32	0.77	0.21	0.50	0.91	0.24	0.44	0.91	0.27	0.46	0.77	0.21
Ni	0.11	0.26	0.06	0.22	0.33	0.02	0.10	0.33	0.02	0.20	0.26	0.06
*Pb*	0.01	0.05	0.01	0.04	0.07	0.00	0.02	0.05	0.00	0.03	0.07	0.00
*Rb*	18.78^a^	20.14	17.28	15.95b	21.43	14.76	17.83	20.04	15.34	17.28	21.43	14.76
Zn	16.24^b^	18.99	14.80	33.70^a^	38.82	27.58	25.65	38.82	14.80	27.58	37.95	15.29

Elements given in italics have been derived from ICP-MS measurements, all other elements were analysed by ICP-OES method. Values with different superscripts differ significantly (Mann-Whitney *U* test) from each other at *p* < 0.05

Interestingly, the thigh muscle has higher (*p* < 0.05, Table 3) levels of Ca, Na, Fe, Mn, and Zn compared with their amounts in the breast muscle. Also, the thigh muscle had lower amounts of Mg, P, and Rb compared with their levels in the breast muscle. These results may indicate some differences between carcass parts in the nutritive value in terms of chemical element content. Similarly, Weig et al. (2016) had found significantly higher levels of Zn, Fe, and Mn in the chicken leg muscle than in the breast muscle. The possible reason for the differences between the thigh and breast meat in the element composition might be, at least in a part, due to the differences in blood perfusion and blood carries, for example, iron and other trace elements. Zinc involved in most of metabolic pathways in human body and its deficiency may result in loss of appetite, impairment of growth in addition to skin, and immunological problems (Weig et al. 2016). Iron is an essential mineral for human life and diet and sufficient iron in the diet is necessary to reduce the occurrence of anaemia (Ghaedi et al. 2006). Copper is vital for many biological systems and was shown to be higher in the thigh muscle (0.07) of a chicken than the breast muscle (0.04 mg/100 g weight; Ferreira et al. 2005).

Taken together, DON contamination of broiler feed did not change the concentration of either micro elements or macro elements of muscles under our experimental conditions.
